# Trends in Emergency Department Visits and Hospital Admissions in Health Care Systems in 5 States in the First Months of the COVID-19 Pandemic in the US

**DOI:** 10.1001/jamainternmed.2020.3288

**Published:** 2020-08-03

**Authors:** Molly M. Jeffery, Gail D’Onofrio, Hyung Paek, Timothy F. Platts-Mills, William E. Soares, Jason A. Hoppe, Nicholas Genes, Bidisha Nath, Edward R. Melnick

**Affiliations:** 1 Department of Emergency Medicine, Mayo Clinic, Rochester, Minnesota; 2 Department of Health Care Policy Research, Mayo Clinic, Rochester, Minnesota; 3 Department of Emergency Medicine, Yale University School of Medicine, New Haven, Connecticut; 4 Information Technology Services, Yale New Haven Health System, New Haven, Connecticut; 5 Department of Emergency Medicine, University of North Carolina School of Medicine, Chapel Hill; 6 Department of Emergency Medicine, University of Massachusetts Medical School–Baystate, Springfield; 7 Department of Emergency Medicine, University of Colorado, School of Medicine, Aurora; 8 Department of Emergency Medicine, Icahn School of Medicine at Mount Sinai, New York, New York

## Abstract

**Importance:**

As coronavirus disease 2019 (COVID-19) spread throughout the US in the early months of 2020, acute care delivery changed to accommodate an influx of patients with a highly contagious infection about which little was known.

**Objective:**

To examine trends in emergency department (ED) visits and visits that led to hospitalizations covering a 4-month period leading up to and during the COVID-19 outbreak in the US.

**Design, Setting, and Participants:**

This retrospective, observational, cross-sectional study of 24 EDs in 5 large health care systems in Colorado (n = 4), Connecticut (n = 5), Massachusetts (n = 5), New York (n = 5), and North Carolina (n = 5) examined daily ED visit and hospital admission rates from January 1 to April 30, 2020, in relation to national and the 5 states’ COVID-19 case counts.

**Exposures:**

Time (day) as a continuous variable.

**Main Outcomes and Measures:**

Daily counts of ED visits, hospital admissions, and COVID-19 cases.

**Results:**

A total of 24 EDs were studied. The annual ED volume before the COVID-19 pandemic ranged from 13 000 to 115 000 visits per year; the decrease in ED visits ranged from 41.5% in Colorado to 63.5% in New York. The weeks with the most rapid rates of decrease in visits were in March 2020, which corresponded with national public health messaging about COVID-19. Hospital admission rates from the ED were stable until new COVID-19 case rates began to increase locally; the largest relative increase in admission rates was 149.0% in New York, followed by 51.7% in Massachusetts, 36.2% in Connecticut, 29.4% in Colorado, and 22.0% in North Carolina.

**Conclusions and Relevance:**

From January through April 2020, as the COVID-19 pandemic intensified in the US, temporal associations were observed with a decrease in ED visits and an increase in hospital admission rates in 5 health care systems in 5 states. These findings suggest that practitioners and public health officials should emphasize the importance of visiting the ED during the COVID-19 pandemic for serious symptoms, illnesses, and injuries that cannot be managed in other settings.

## Key Points

**Question:**

How did emergency department visits and hospitalizations change as the coronavirus disease 2019 (COVID-19) pandemic intensified in the US?

**Findings:**

In this cross-sectional study of 24 emergency departments in 5 health care systems in Colorado, Connecticut, Massachusetts, New York, and North Carolina, decreases in emergency department visits ranged from 41.5% in Colorado to 63.5% in New York, with the most rapid rates of decrease in visits occurring in early March 2020. Rates of hospital admissions from the ED were stable until new COVID-19 case rates began to increase locally, at which point relative increases in hospital admission rates ranged from 22.0% to 149.0%.

**Meaning:**

The findings suggest that clinicians and public health officials should emphasize to patients the importance of continuing to visit the emergency department for serious symptoms, illnesses, and injuries that cannot be managed in other clinical settings.

## Teaser

This cross-sectional study examines trends in emergency department visits and visits that led to hospitalizations during a 4-month period leading up to and during the COVID-19 outbreak in the US.

## Introduction

As coronavirus disease 2019 (COVID-19) spread throughout the US in the early months of 2020, the delivery of acute care changed to accommodate an influx of patients with a highly contagious infection about which little was known. Initial public health messaging advised avoiding unnecessary health care use to reduce transmission of the virus and to ensure capacity to accommodate surges in COVID-19 cases.^1^ An early report^2^ suggested that use of health care services for elective and emergency conditions decreased during this period. Reductions in emergency department (ED) use could reflect (1) failure by patients with serious or life-threatening conditions to seek care, including conditions unrelated to COVID-19^3^; (2) avoidance of the ED for nonemergency conditions; or (3) displacement of ED care to other venues, such as telemedicine visits.^4^ We studied changes in ED use in 5 health care systems representing geographically diverse areas in 5 states in the first months of the COVID-19 pandemic in the US.

## Methods

This cross-sectional study used data from a number of large US health care systems that were collected as part of an ongoing trial of ED prescribing practices for opioid use disorder; the original study protocol was approved by the Western Institutional Review Board with reliance agreements by the individual institutions’ institutional review boards.^5^ The Western Institutional Review Board approved an amendment to this study protocol with an exemption of informed consent to collect data on ED visits and hospital admissions to better understand the association of COVID-19 with trial enrollment. The study also used deidentified quality improvement data from Mount Sinai Health System (New York City) that were collected to assess staffing and resource use during the COVID-19 outbreak and were considered exempt from institutional review board review under 45 CFR §46.101(b)(4).

For January 1 to April 30, 2020, we examined trends in daily ED visits and the rate of hospital admissions from EDs that are part of 5 large, independent health care systems in 5 states. One data set came from Mount Sinai Health System (New York), and four came from health systems in the EMBED trial: Baystate Health (Massachusetts), University of Colorado Health (UCHealth, Colorado), Mount Sinai Health (New York), University of North Carolina (UNC) Health, and Yale New Haven Health (Connecticut). We analyzed these trends in the context of publicly reported national and state COVID-19 case counts. We abstracted visit data from electronic health record databases at each health care system with structured queries of their local Epic Clarity databases (Epic Systems), with the exception of Baystate Health, which uses Cerner (Cerner Corporation). We retrieved daily COVID-19 case counts from the Johns Hopkins University Center for Systems Science and Engineering public data feed.^6^ We standardized new confirmed state COVID-19 cases to state populations using US Census Bureau data on estimated population as of July 1, 2019.^7^

We display the data as scatterplots with overlaid nonparametric smoothed curves generated with a locally weighted scatterplot smoothing (LOWESS; bandwidth 0.2) method. This method computes a separate least-squares regression for each data point, using a subset of points around the central data point in the regression and applying greater statistical weight to nearer points.^8^ In addition to being a useful visualization technique, LOWESS can be used to estimate fitted values of the dependent variable for each value of the independent variable. These values were used to compute relative changes in admission rates and case counts to minimize the effect of outliers by estimating minima and maxima based on a local weighted mean, rather than using the more extreme observed maxima and minima; relative changes were calculated based on the LOWESS-estimated extrema. All analyses were performed using Stata statistical software, version 16.1 (StataCorp).

## Results

The 24 EDs varied widely in size and setting; data came from 5 EDs in Connecticut, Massachusetts, New York, and North Carolina and 4 EDs in Colorado. Annual ED volume before the COVID-19 pandemic ranged from 13 000 to 115 000 visits per year; 15 of the EDs were in urban areas, 5 were suburban, and 4 were rural; 7 were academic and 17 were community sites (Table).

**Table.** Health Care System Site Characteristics^a^

Site	Location	2019 Annual ED patient volume, visits per year	2019 ED admissions per year	Academic site	Geographic classification
Baystate Health					
Franklin	Greenfield, Massachusetts	28 000	4900	No	Rural
Mary Lane	Ware, Massachusetts	13 000	0^b^	No	Urban
Noble	Westfield, Massachusetts	28 000	3000	No	Urban
Springfield	Springfield, Massachusetts	115 000	42 400	Yes	Urban
Wing	Palmer, Massachusetts	22 000	3300	No	Urban
UCHealth					
Anschutz Medical Campus	Aurora, Colorado	103 000	19 500	Yes	Urban
Medical Center of the Rockies	Loveland, Colorado	56 000	13 400	No	Suburban
Memorial Central	Colorado Springs, Colorado	109 000	20 700	No	Urban
Poudre Valley	Fort Collins, Colorado	65 000	11 000	No	Suburban
Mount Sinai Health					
Mount Sinai Brooklyn	Brooklyn, New York	37 000	9100	No	Urban
Mount Sinai Hospital	New York, New York	107 000	19 600	Yes	Urban
Mount Sinai Morningside	New York, New York	89 000	11 800	Yes	Urban
Mount Sinai Queens	Queens, New York	69 000	10 000	No	Urban
Mount Sinai West	New York, New York	70 000	9000	Yes	Urban
UNC Health					
Chatham	Siler City, North Carolina	17 800	1000	No	Rural
Johnston-Smithfield	Smithfield, North Carolina	43 000	6500	No	Rural
Memorial	Chapel Hill, North Carolina	67 000	21 200	Yes	Suburban
Nash	Rocky Mount, North Carolina	70 000	11 700	No	Rural
Rex	Raleigh, North Carolina	68 000	19 400	No	Urban
Yale–New Haven Health					
Bridgeport	Bridgeport, Connecticut	93 000	15 900	No	Urban
Greenwich	Greenwich, Connecticut	38 000	8000	No	Suburban
Lawrence & Memorial	New London, Connecticut	49 000	10 600	No	Suburban
Saint Raphael Campus	New Haven, Connecticut	65 000	18 600	Yes	Urban
York Street Campus	New Haven, Connecticut	96 000	27 900	No	Urban

Abbreviations: ED, emergency department; UCHealth, University of Colorado Health; UNC, University of North Carolina.

^a^ Per convention, visit volume data are reported to the nearest thousand and were abstracted from electronic health record databases at each health care system by applying structured query language similarly to the data for the primary analysis.

^b^ All ED admissions from Baystate Mary Lane are admitted to Baystate Wing and are included in the Wing admission number.

**Figure 1.**
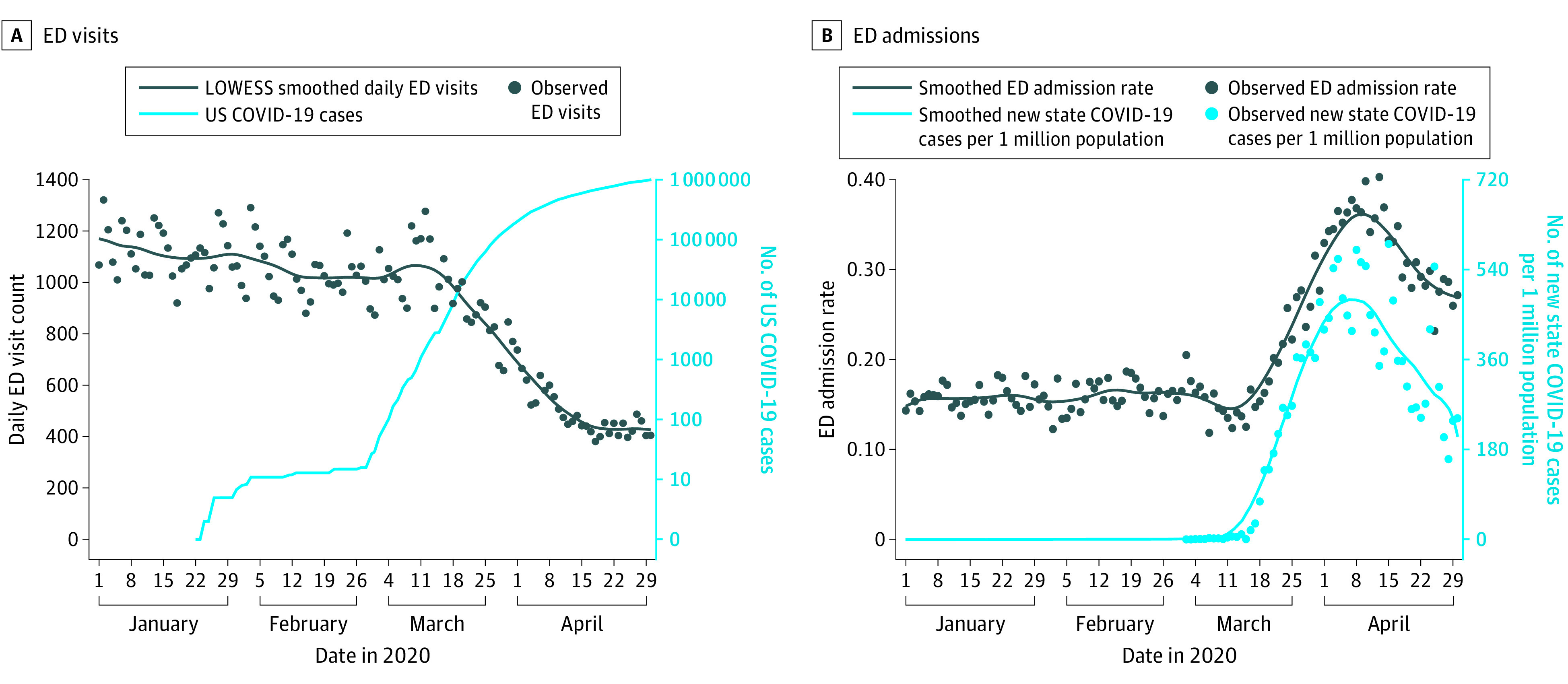
Daily Emergency Department (ED) Visits and Admissions to the Mount Sinai Health System from January 1 through April 30, 2020 A, Emergency department visit counts in 5 EDs in New York and US coronavirus disease 2019 (COVID-19) cases (plotted on a log scale) are shown. B, Hospital admission rates from the ED and New York’s new daily confirmed COVID-19 cases per 1 million population are shown. New York data are plotted separately to avoid obscuring trends in states with lower daily ED visit counts.

**Figure 2.**
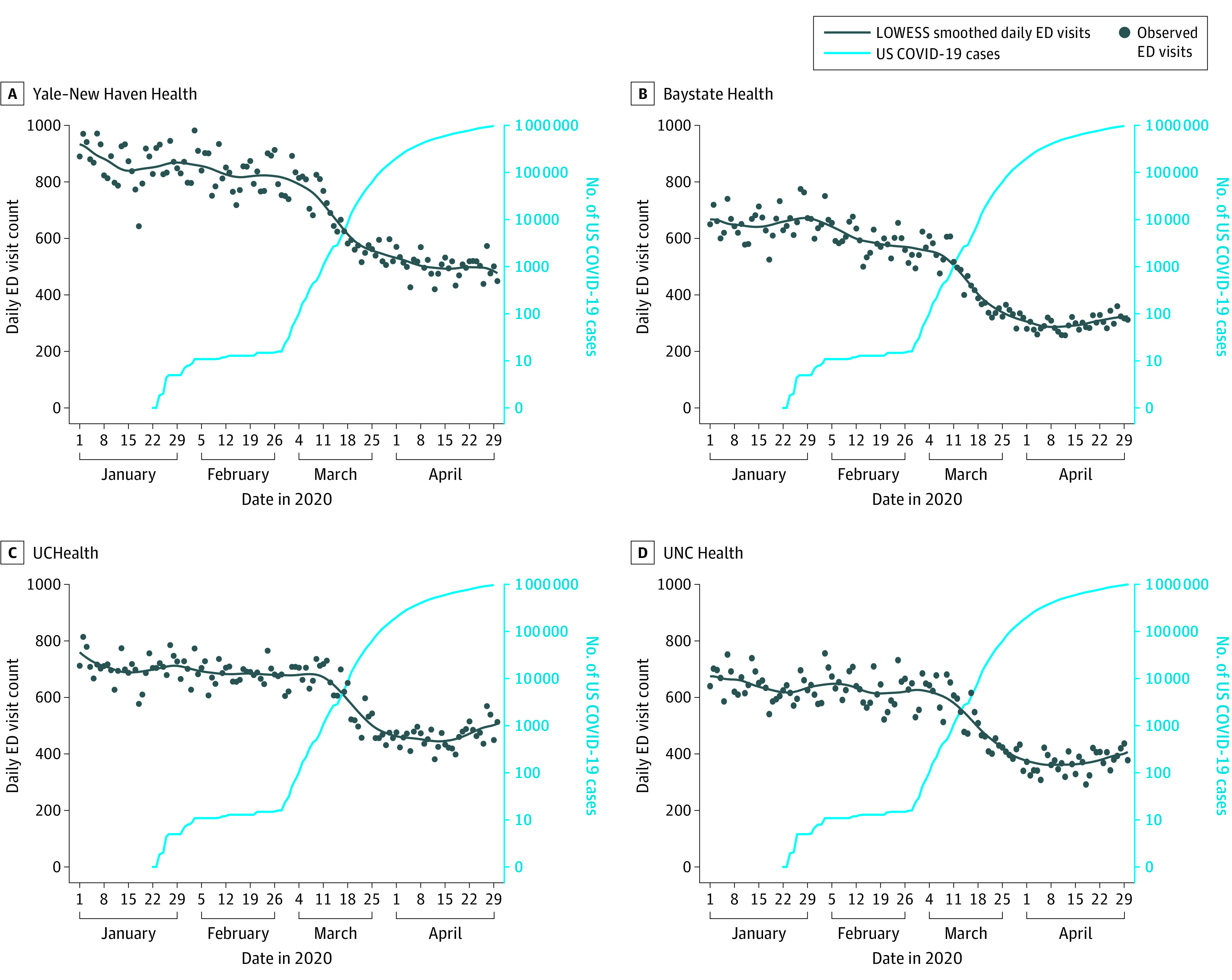
Daily Emergency Department (ED) Visits in 4 Health Systems in 4 States from January 1 through April 30, 2020 Emergency department visit counts in 19 EDs in 4 states and US coronavirus disease 2019 cases (plotted on a log scale) are shown. Circles indicate specific daily values for each variable. UCHealth indicates University of Colorado Health; UNC, University of North Carolina.

**Figure 3.**
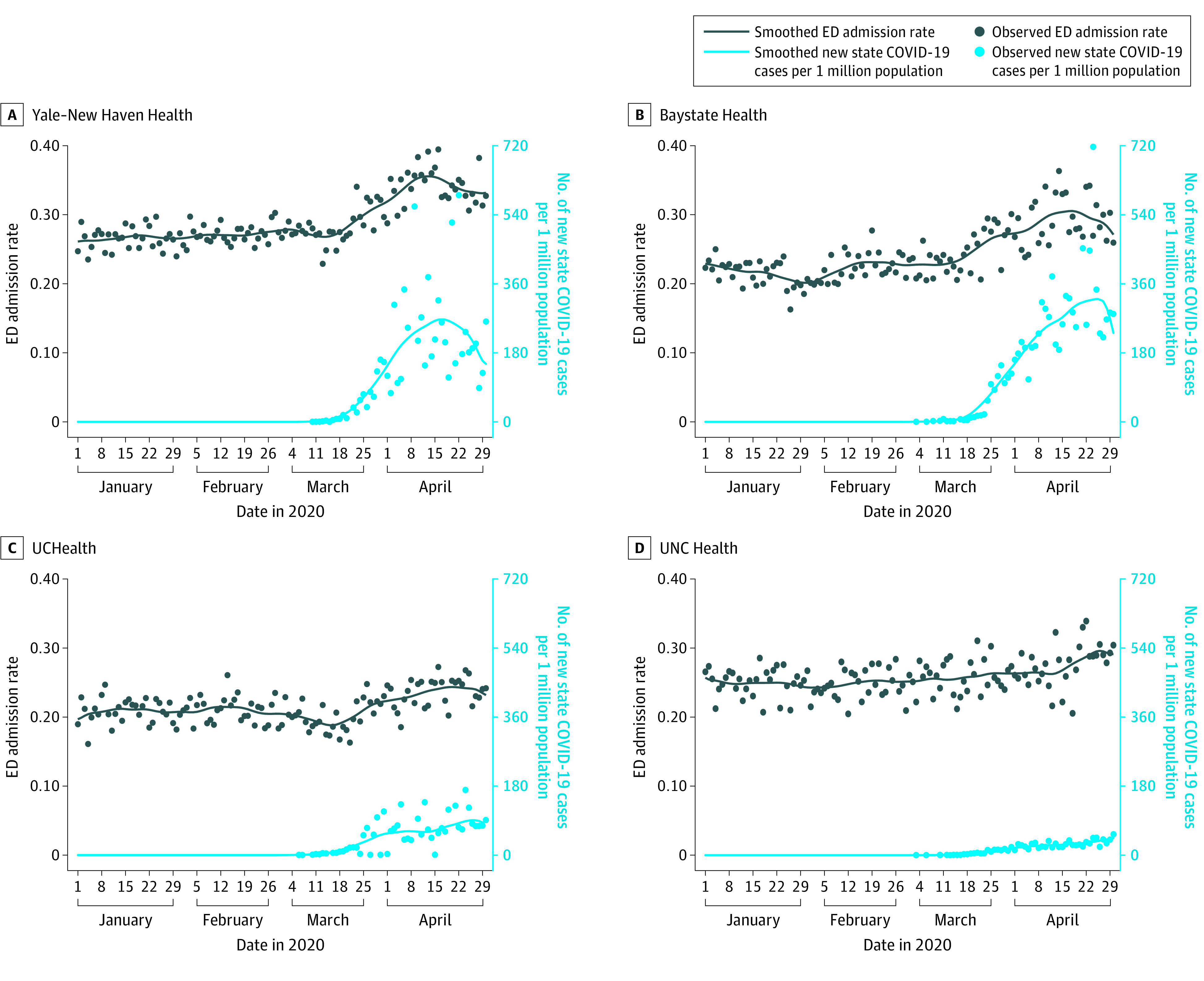
Daily Emergency Department (ED) Hospitalizations in 4 Health Systems in 4 States from January 1 through April 30, 2020 Hospital admission rates from the ED in 4 states and each state’s new daily confirmed coronavirus disease 2019 cases per 1 million population are shown. Circles indicate specific daily values for each variable. UCHealth indicates University of Colorado Health; UNC, University of North Carolina.

In all 5 states, there were large decreases in ED visits, with the most rapid decrease beginning the week of March 11, 2020, as the increase in the US case count for COVID-19 accelerated (Figure 1 and Figure 2). The largest decrease in LOWESS estimates of visits was seen in New York (63.5%), followed by Massachusetts (57.4%), Connecticut (48.9%), North Carolina (46.5%), and Colorado (41.5%). In 3 states, (Massachusetts, Colorado, and North Carolina), small increases in ED visits occurred in late April 2020. Trends in rates of hospital admissions from the ED were associated with state-level new COVID-19 case counts (Figure 1 and Figure 3). Hospital admission rates were stable in each state until that state’s COVID-19 case rate began to increase. The largest relative increase in LOWESS estimates of admission rates was 149.0% in New York, followed by 51.7% in Massachusetts, 36.2% in Connecticut, 29.4% in Colorado, and 22.0% in North Carolina.

Hospital admission rates were initially steady despite decreasing ED visits because trends in hospital admission counts were associated with ED visit counts (eFigures 1-5 in the Supplement). The temporal association between the increase in each state’s COVID-19 caseload and hospital admission rates was less apparent when hospital admission counts were plotted. The exception is in the Mount Sinai Health System, where smoothed hospital admission counts appeared to peak approximately 1 week before the peak of the New York COVID-19 case rate. The ED visit count in the Mount Sinai Health System continued to decrease as hospital admission counts and state COVID-19 cases increased; there was a similar although less pronounced pattern for Yale New Haven Health.

## Discussion

As the COVID-19 pandemic developed and intensified in the US during the first 4 months of 2020, we found that ED visit counts decreased and the rates of hospital admissions from the ED increased in 5 health care systems in 5 states. From their height in January to their lowest point in April, ED visits decreased by more than 40% in all the health care systems and by more than 60% in New York, where the pandemic was most severe. Rates of hospital admission from the ED were stable until COVID-19 cases increased locally, suggesting lower patient volume and higher acuity in the ED as the COVID-19 pandemic spread. Despite different timing and increased rates of COVID-19 cases locally, we observed similar patterns and timing of ED visits across the 5 health care systems, with the steepest decrease in visits beginning the week of March 11, 2020. A possible explanation for these temporal associations is that the public responded more to national-level risk messaging about COVID-19 than to changes in the local situation with regard to reported cases. For example, individuals may have avoided seeking emergency care because of a fear of being exposed to COVID-19 in the ED, concerns about the possibility of extended wait times, or a sense of civic responsibility to avoid using health care services that others may have needed.^2^

Even as ED visits decreased most rapidly, initial admission rates from the ED were initially stable, indicating that admission counts were decreasing as well. However, a temporal association was found between the increase in each state’s COVID-19 caseload and admission rates. We did not attempt to identify ED visits possibly associated with COVID-19, so we cannot report the decrease in non–COVID-19 ED visits. The association between COVID-19 and ED visits by patients seeking care for reasons unrelated to COVID requires further study.

Although our study could not establish the reasons for the changes in ED visits and hospital admissions that we observed, it provides insight into the COVID-19 pandemic for the medical community and the public during the COVID-19 pandemic. First, practitioners and public health officials should emphasize the importance of continuing to visit the ED for serious symptoms, illnesses, and injuries that cannot be managed in other settings, such as telemedicine visits. Second, infection control measures that protect patients and staff are essential in the ED and other clinical settings. Third, public health authorities and health care systems should provide guidance and resources to help patients determine the best place to receive care as the available health care capacity changes during the pandemic.^9^

### Limitations

Among the limitations of our study is that the findings may not be generalizable outside the 5 health care systems that we studied. Moreover, the study data did not include diagnoses; therefore, we could not assess how the ED patient case mix may have changed during the study period. Although the data revealed a steep decrease in the use of the ED in the 5 health care systems from the middle of March to the middle of April 2020, they cannot be used to determine whether people with serious symptoms, illnesses, and injuries went untreated because of the COVID-19 pandemic.^10^

## Conclusions

From January through April 2020, as the COVID-19 pandemic intensified in the US, temporal associations were observed with a decrease in ED visits and an increase in hospital admission rates in 5 health care systems in 5 states. These findings suggest that practitioners and public health officials should emphasize the importance of visiting the ED during the COVID-19 pandemic for serious symptoms, illnesses, and injuries that cannot be managed in other settings.

## Notes

Supplement. **eFigure 1.** Daily Emergency Department Visit and Admission Counts in 5 EDs in NY **eFigure 2.** Daily Emergency Department Visit and Admission Counts in 5 EDs in CO **eFigure 3.** Daily Emergency Department Visit and Admission Counts in 4 EDs in CT **eFigure 4.** Daily Emergency Department Visit and Admission Counts in 5 EDs in MA **eFigure 5.** Daily Emergency Department Visit and Admission Counts in 5 EDs in NC
