# Supplementary material for: Trends in Emergency Department Visits and Hospital Admissions in Health Care Systems in 5 States in the First Months of the COVID-19 Pandemic in the US
Source: JAMA Intern Med. 2020 Aug 3;180(10):1328–33. doi: 10.1001/jamainternmed.2020.3288 (PMC7400214; doi:10.1001/jamainternmed.2020.3288)
Supplement: Supplement. — eFigure 1. Daily Emergency Department Visit and Admission Counts in 5 EDs in NY eFigure 2. Daily Emergency Department Visit and Admission Counts in 5 EDs in CO eFigure 3. Daily Emergency Department Visit and Admission Counts in 4 EDs in CT eFigure 4. Daily Emergency Department Visit and Admission Counts in 5 EDs in MA eFigure 5. Daily Emergency Department Visit and Admission Counts in 5 EDs in NC [file jamainternmed-e203288-s001.pdf]

Supplementary Online Content

Jeffery MM, D'Onofrio G, Paek H, et al. Trends in emergency department visits and hospital admissions in health care systems in 5 states in the first months of the COVID-19 pandemic in the US. *JAMA Intern Med.* Published online August 3, 2020.
doi:10.1001/jamainternmed.2020.3288

eFigure 1. Daily Emergency Department Visit and Admission Counts in 5 EDs in NY

eFigure 2. Daily Emergency Department Visit and Admission Counts in 5 EDs in CO

eFigure 3. Daily Emergency Department Visit and Admission Counts in 4 EDs in CT

eFigure 4. Daily Emergency Department Visit and Admission Counts in 5 EDs in MA

eFigure 5. Daily Emergency Department Visit and Admission Counts in 5 EDs in NC

This supplementary material has been provided by the authors to give readers additional information about their work.

Open Access: This is an open access article distributed under the terms of the CC-BY License.

eFigure 1. Daily Emergency Department Visit and Admission Counts in 5 EDs in NY

ED visit counts, ED admission counts, and new daily confirmed COVID-19 cases per 1 million population: January 1 through April 30, 2020. Lines represent LOWESS smoothed curves. Scatterplot points represent observed values.

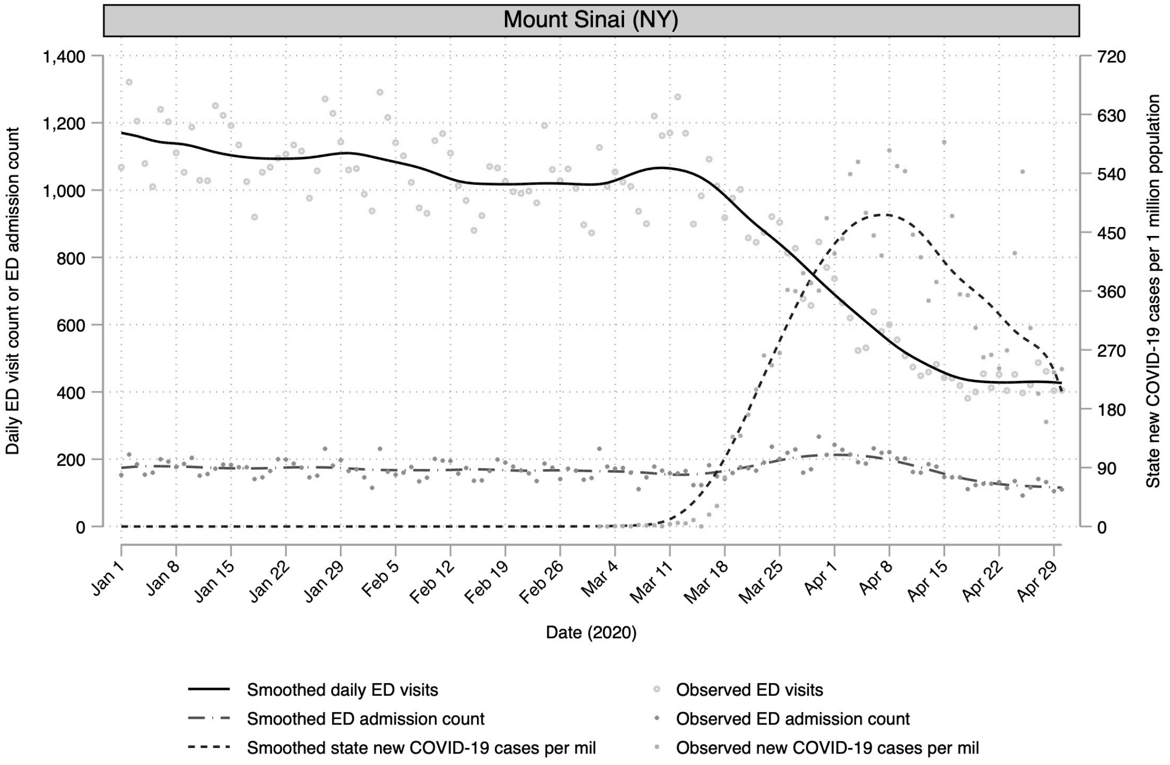

Open Access: This is an open access article distributed under the terms of the CC-BY License.

eFigure 2. Daily Emergency Department Visit and Admission Counts in 5 EDs in CO
ED visit counts, ED admission counts, and new daily confirmed COVID-19 cases per 1 million population: January 1 through April 30, 2020. Lines represent LOWESS smoothed curves. Scatterplot points represent observed values.

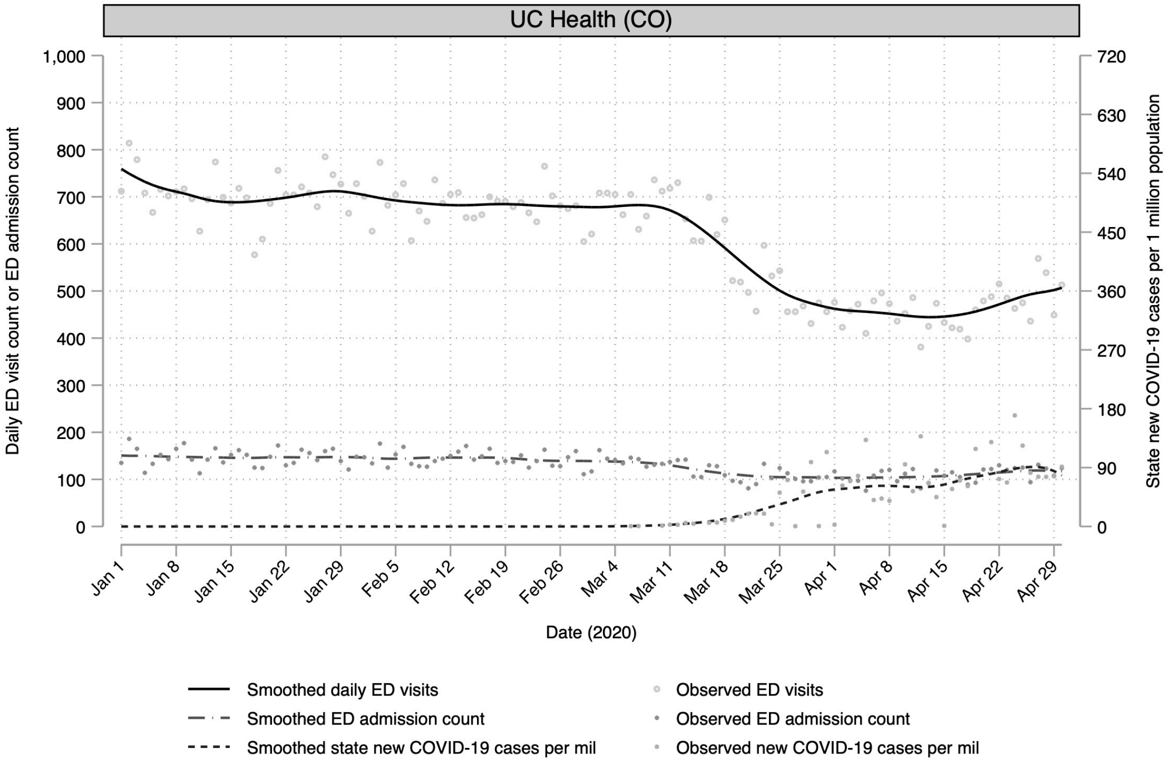

Open Access: This is an open access article distributed under the terms of the CC-BY License.

eFigure 3. Daily Emergency Department Visit and Admission Counts in 4 EDs in CT

ED visit counts, ED admission counts, and new daily confirmed COVID-19 cases per 1 million population: January 1 through April 30, 2020. Lines represent LOWESS smoothed curves. Scatterplot points represent observed values.

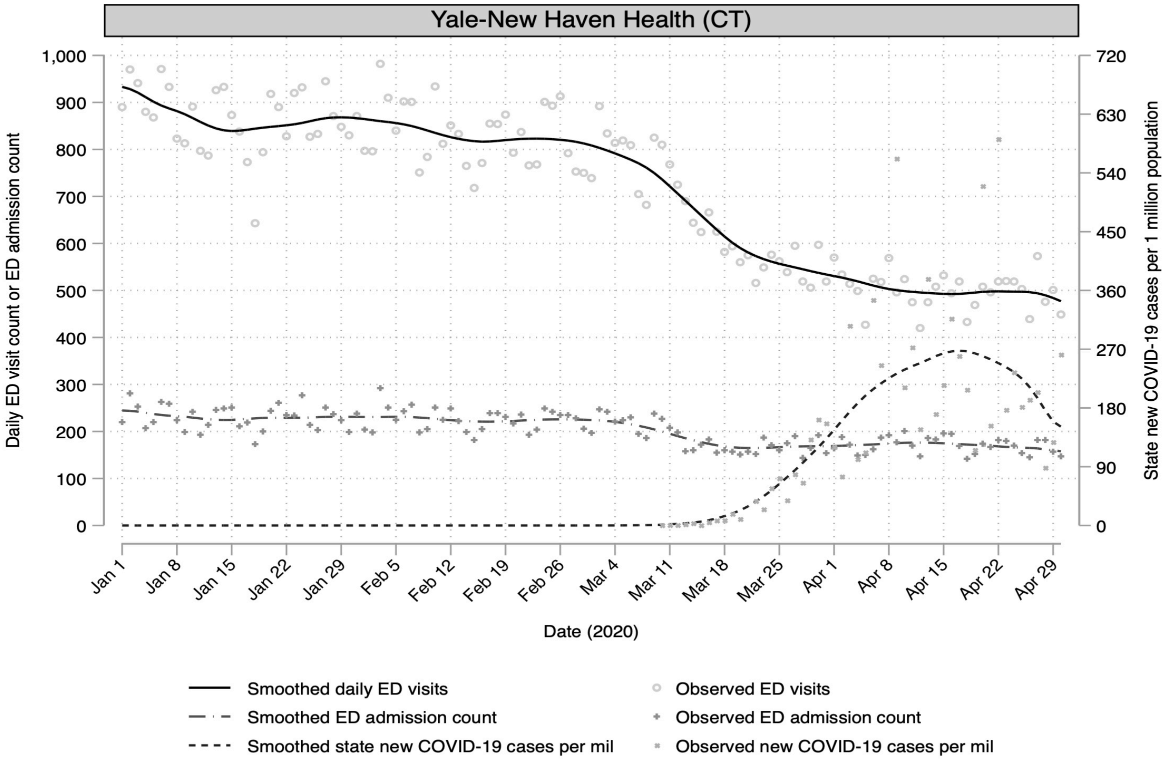

Open Access: This is an open access article distributed under the terms of the CC-BY License.

eFigure 4. Daily Emergency Department Visit and Admission Counts in 5 EDs in MA

ED visit counts, ED admission counts, and new daily confirmed COVID-19 cases per 1 million population: January 1 through April 30, 2020. Lines represent LOWESS smoothed curves. Scatterplot points represent observed values.

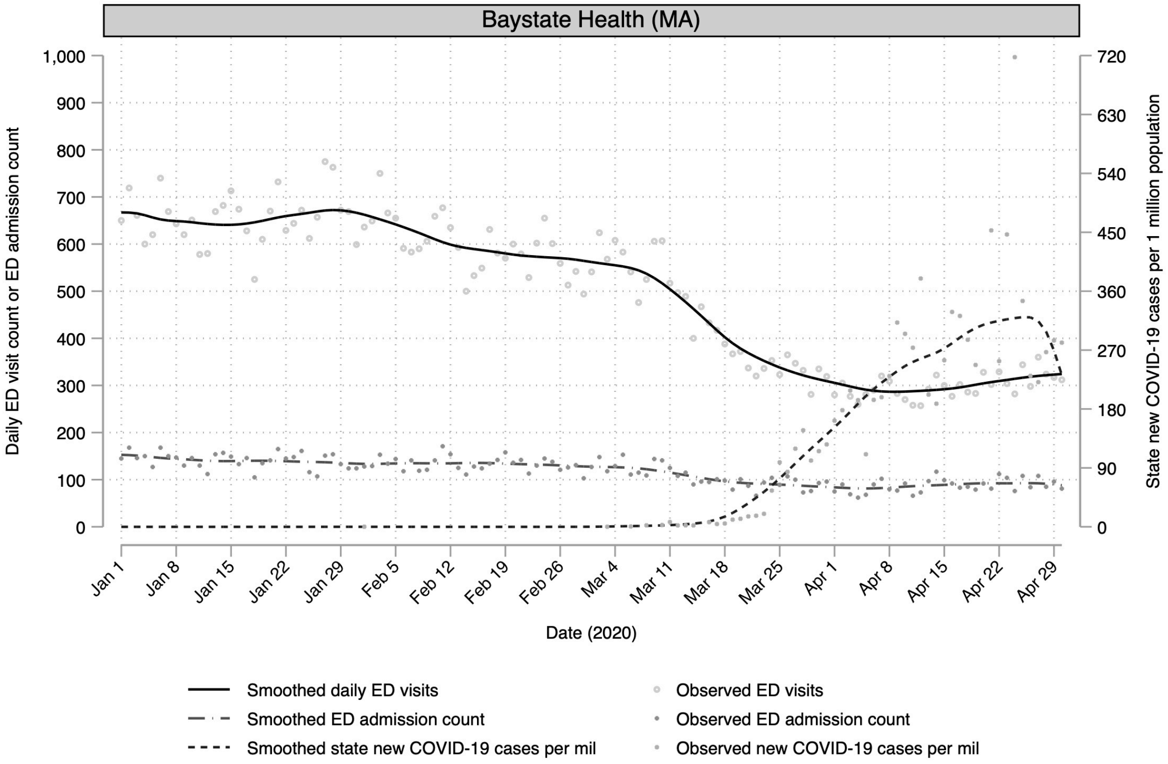

Open Access: This is an open access article distributed under the terms of the CC-BY License.

eFigure 5. Daily Emergency Department Visit and Admission Counts in 5 EDs in NC
ED visit counts, ED admission counts, and new daily confirmed COVID-19 cases per 1 million population: January 1 through April 30, 2020. Lines represent LOWESS smoothed curves. Scatterplot points represent observed values.

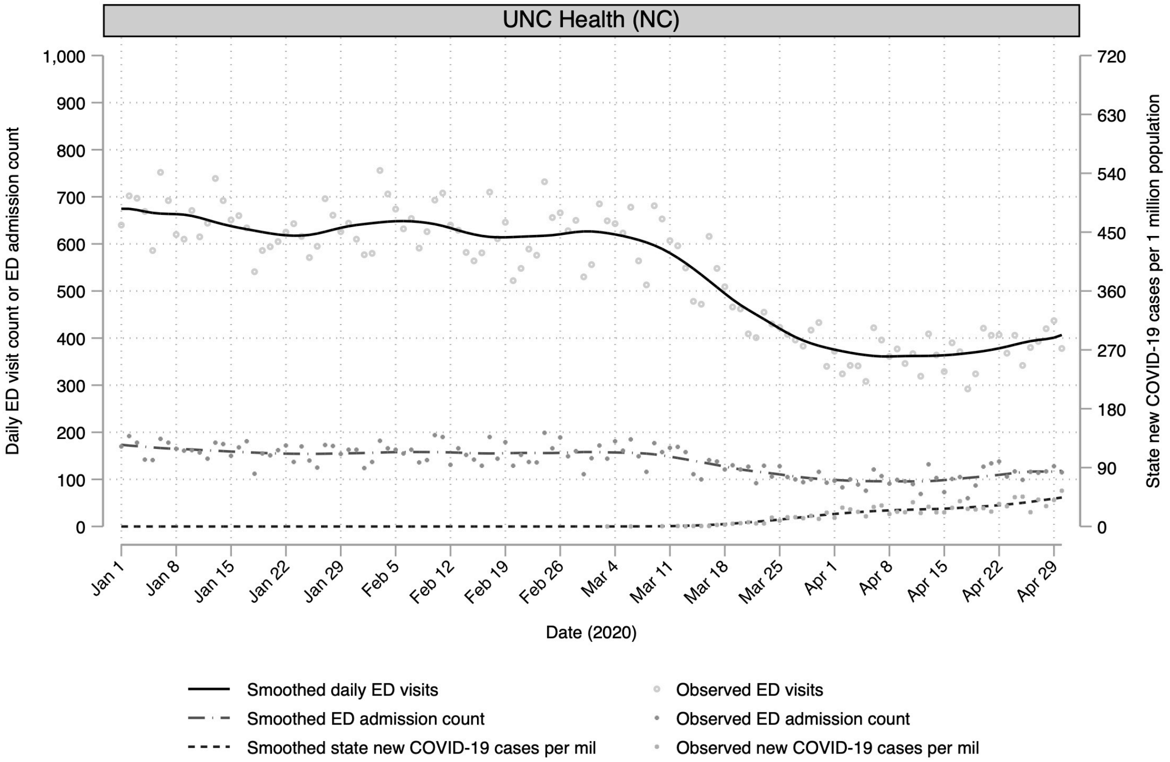

Open Access: This is an open access article distributed under the terms of the CC-BY License.

Supplemental Materials. Daily Emergency Department Visit and Admission Counts in 5 health systems in 5 states ED visit counts, ED admission counts, and new daily confirmed COVID-19 cases per 1 million population: January 1 through April 30, 2020. Lines represent LOWESS smoothed curves. Scatterplot points represent observed values.

Figure 1. Daily Emergency Department Visit and Admission Counts in 5 EDs in CO
Figure 1b. Daily Emergency Department Visit and Admission Counts in 4 EDs in CT
Figure 1c. Daily Emergency Department Visit and Admission Counts in 5 EDs in MA
Figure 1d. Daily Emergency Department Visit and Admission Counts in 5 EDs in NY
Figure 1e. Daily Emergency Department Visit and Admission Counts in 5 EDs in NC

Open Access: This is an open access article distributed under the terms of the CC-BY License.
